# Effect of lung compliance and endotracheal tube leakage on measurement of tidal volume

**DOI:** 10.1186/cc2954

**Published:** 2004-10-06

**Authors:** Sami I Al-Majed, John E Thompson, Kenneth F Watson, Adrienne G Randolph

**Affiliations:** 1 Attending in Pediatric pulmonary and Intensive Care and Director of Pediatric ICU, Dhahran Health Center, Saudi ARAMCO, Saudi Arabia; 2 Director of Respiratory Care and Biomedical Engineering, Children's Hospital Boston, Boston, MA, USA; 3 Coordinator of Clinical Research, Respiratory Care Department, Children's Hospital Boston, Boston, MA, USA; 4 Associate Professor of Anesthesia (Pediatrics), Harvard Medical School, Boston, MA, USA, Director of Patient Safety and Quality Improvement, Medical-Surgical ICU & Senior Associate in Critical Care, Department of Anesthesia, Children's Hospital Boston, Boston, MA, USA

**Keywords:** intensive care, lung compliance, mechanical ventilation, monitoring tidal volume

## Abstract

**Introduction:**

The objective of this laboratory study was to measure the effect of decreased lung compliance and endotracheal tube (ETT) leakage on measured exhaled tidal volume at the airway and at the ventilator, in a research study with a test lung.

**Methods:**

The subjects were infant, adult and pediatric test lungs. In the test lung model, lung compliances were set to normal and to levels seen in acute respiratory distress syndrome. Set tidal volume was 6 ml/kg across a range of simulated weights and ETT sizes. Data were recorded from both the ventilator light-emitting diode display and the CO_2_SMO Plus monitor display by a single observer. Effective tidal volume was calculated from a standard equation.

**Results:**

In all test lung models, exhaled tidal volume measured at the airway decreased markedly with decreasing lung compliance, but measurement at the ventilator showed minimal change. In the absence of a simulated ETT leak, calculation of the effective tidal volume led to measurements very similar to exhaled tidal volume measured at the ETT. With a simulated ETT tube leak, the effective tidal volume markedly overestimated tidal volume measured at the airway.

**Conclusion:**

Previous investigators have emphasized the need to measure tidal volume at the ETT for all children. When ETT leakage is minimal, it seems from our simulated lung models that calculation of effective tidal volume would give similar readings to tidal volume measured at the airway, even in small patients. Future studies of tidal volume measurement accuracy in mechanically ventilated children should control for the degree of ETT leakage.

## Introduction

Three investigators have reported that tidal volume (*V*_T_) in children is inaccurate when measured at the ventilator, even when effective *V*_T _is used [1-3]. Cannon and colleagues [1] studied 98 infants and children and found a significant discrepancy between expiratory *V*_T _measured at the ventilator and that measured with a pneumotachometer.

Calculation of the effective *V*_T _did not alter this discrepancy. Castle and colleagues [2] studied 56 intubated children and found that exhaled *V*_T _displayed by the Servo 300 significantly overestimated *V*_T _measured at the airway by between 2% and 91%. After correcting for gas compression, effective *V*_T _overestimated true *V*_T _by as much as 29% in older children but underestimated the true *V*_T _by up to 64% in the smallest infants. Neve and colleagues [3] studied 27 infants and found that *V*_T _was overestimated by the ventilator in comparison with *V*_T _measured at the Y piece. None of these investigators controlled for endotracheal tube (ETT) leakage, which is more of a problem in children than in adults because of the use of uncuffed ETTs.

Accurate measurement of *V*_T _is increasingly important because the Acute Respiratory Distress Syndrome (ARDS) Network investigators have shown that the use of a low effective *V*_T _leads to decreased mortality in their patient population [4]. The effective *V*_T _goal in their ventilator protocol was 6 ml/kg but could be reduced to as low as 4 ml/kg if the plateau pressure was above 30 cmH_2_O. At such low *V*_T _values, accurate measurement is imperative to prevent atelectasis and subsequent ineffective minute ventilation.

Clinically, there are three methods to estimate delivered *V*_T_: first, direct measurement at the expiratory limb of the ventilator; second, direct measurement at the ETT with a pneumotachometer; and third, indirect calculation of effective *V*_T _by using set *V*_T _minus calculated compressible volume lost in the ventilator circuit [5]. The principle of Boyle's law (the volume of gas decreases as the absolute pressure exerted by the gas increases, and vice versa) is used to calculate the compressible volume in ventilator circuits.

How effective *V*_T _compares with *V*_T _measured at the airway has not been rigorously tested. Using *V*_T _measured at the ETT as the gold standard, we used three test lung models in a controlled laboratory setting to evaluate the accuracy of ventilator measured *V*_T _and effective *V*_T _under conditions of poor lung compliance, with and without ETT leakage, across a range of simulated patient sizes. We proposed that the discrepancy between effective *V*_T _and *V*_T _measured at the ETT in children was due mainly to ETT leakage around uncuffed ETTs, and that in situations with minimal ETT leakage there would be minimal difference between the effective *V*_T _and *V*_T _measured at the airway.

## Materials and methods

### Experimental conditions

A Servo 300 ventilator (Siemens-Elema, Solna, Sweden) in the SIMV volume control mode was used. A pressure differential pneumotachometer (CO_2_SMO Plus; Novametrix Medical Systems, Wallingford, CT) was used between the ventilator and ETT connection. The temperature of the humidifier was set at 37°C. A heated disposable respiratory circuit (Allegiance Healthcare Corporation, McGaw Park, IL) was used. We tested the compliance of the circuit to ensure that it was stable across a range of conditions. To do this, we first set the ventilator on the following: inspiratory time of 1.3 s, positive end-expiratory pressure (PEEP) of 0, respiratory set frequency of 6 breaths per minute, and a pause time of 15%. *V*_T _was increased by increments of 50 ml and the plateau pressure was recorded from the ventilator with the patient outlet occluded. No component other than the humidifier was added to the circuit [6]. A linear relationship was found, with no change of the circuit compliance at high airway pressure.

**Figure 1:**
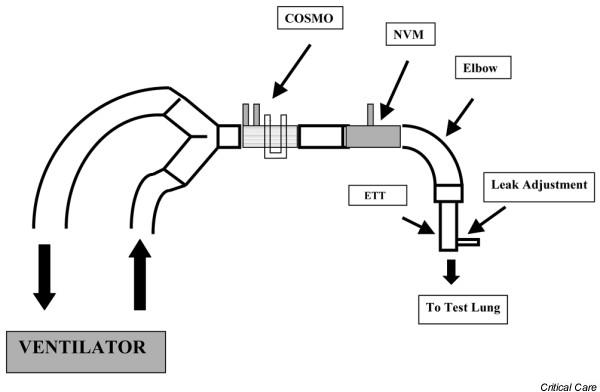
Schematic diagram demonstrating the placement of CO_2_SMO and NMV pnueumotachometers in infant and pediatric models.

In the pediatric and infant models, a valve distal to the ETT was used to adjust volume leaks of 0%, 10%, 20%, and 30%. A shown in Fig. 1, a separate pneumotachometer (NVM-1; Thermo Respiratory Group, Palm Springs, CA) was used for independent measurement of the percentage of ETT leakage.

The Servo 300 was used for all test conditions. To control for differences between the ventilators, we tested each set of experimental conditions on three different ventilators. The CO_2_SMO Plus respiratory mechanics monitor was used to measure the *V*_T _at the ETT. This monitor measures flow with a fixed-orifice differential pressure pneumotachometer located at the ETT. Respired gas flowing through the flow sensor produces a small pressure decrease across the two tubes connected to the sensor. This pressure decrease is transmitted through the tubing sensor to a differential pressure transducer inside the monitor and is correlated with flow according to a factory-stored calibration. The pressure transducer is automatically 'zeroed' to correct for changes in ambient temperature. Data are filtered and sampled at 100 Hz. The monitor continuously displays a range of ventilatory variables, including both *V*_T _and airway pressures. Three CO_2_SMO Plus sensors are available: neonatal, pediatric, and adult. The manufacturer recommends that the choice of sensor be based on various criteria: first, the diameter of the tracheal tube; second, the patient's age; third, the expected flow/volume range; and fourth, the acceptable levels of dead space and resistance. Table 1 lists the experimental conditions for all lung models. Before data collection, all ventilators, respiratory mechanics monitors, and tachometers used in this study were calibrated in accordance with the manufacturer's recommendation.

To ensure that different ventilators and monitors did not influence the results, all data were repeated three times, each time with a different Servo 300 ventilator and a different CO_2_SMO Plus monitor.

#### Adult lung model

A TTL™ adult test lung (Vent Aid; Michigan Instruments Inc., Grand Rapids, MI) was used. This device has two separate lungs, each with a functional residual capacity (FRC) of 900 ml. The lung compliance can be adjusted by moving a spring up and down with a compliance ranging from 10 to 150 ml/cmH_2_O per lung. Each lung is tested before use to assess for leakage. Lung–thorax compliance levels were set at 10, 20, 40, 60, 100, and 150 ml/cmH_2_O.

#### Pediatric lung model

A TTL™ adult test single lung was used with the FRC adjusted to give 30 ml/kg by displacing the extra volume with water-filled bags. Lung–thorax compliance levels were set at 5, 10, 20, 40 and 60, ml/cmH_2_O.

#### Infant lung model

An infant lung simulator (D.B&M products, Redlands, CA) was used. The model has three different preset compliances of 1, 3, and 10 ml/cmH_2_O.

### Data recording

Data were recorded from both the ventilator light-emitting diode display and the CO_2_SMO Plus monitor display by a single observer. Variables recorded were inspired *V*_T_, expired *V*_T_, peak inspiratory pressure (PIP), PEEP, and plateau pressure. Effective *V*_T _was calculated from the following equation [2]: set inspired *V*_T _- [circuit compliance × (PIP - PEEP)].

### Analysis

The major outcome variable was the calculated difference between the effective *V*_T _and the exhaled *V*_T _measured either at the ventilator or at the ETT in each experiment. For each set of test conditions (Table 1) we used the mean of the three replicate measurements and also give the highest and lowest values. *V*_T _was adjusted for the simulated weights and expressed as ml/kg. We determined a priori that the difference between the *V*_T _values would be considered excessive if it exceeded 10% of the 6 ml/kg goal (0.6 ml/kg).

## Results

### Test lung models

**Figure 2:**
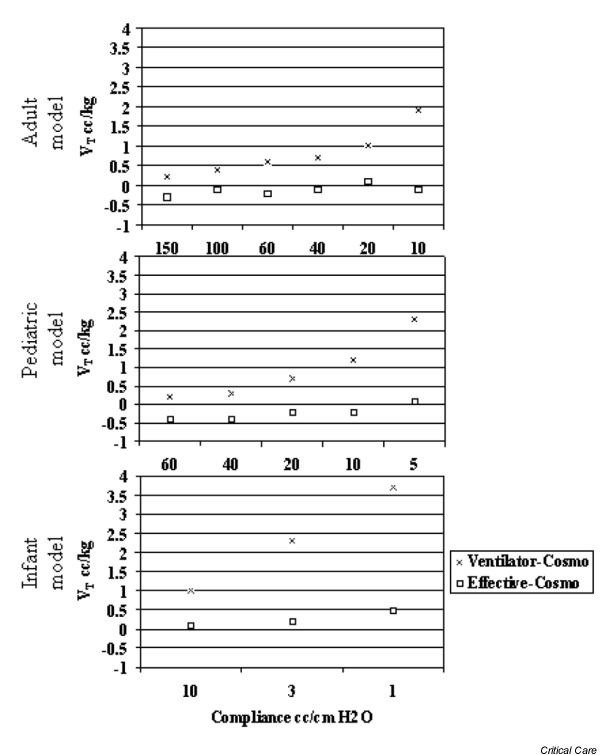
Effect of decreasing lung compliance on the difference between effective tidal volume and tidal volume at the endotracheal tube (ETT) in the infant, pediatric, and adult test lungs with no leak around the ETT.

As shown in Fig. 2, for the adult, pediatric, and infant models with no ETT leak, the difference between *V*_T _measured at the ETT and at the ventilator increased with decreasing lung compliance. *V*_T _measured at the ventilator was always higher than that measured at the ETT. The ventilator measurement overestimated *V*_T _by more than 10% (0.6 ml/kg) as lung compliance dropped to moderately low values and the difference exceeded 20% (1.8 ml/kg) at the lowest lung compliances in each model. The standard deviation of the difference was 0–0.2 ml/kg for all sets of measurements.

**Figure 3:**
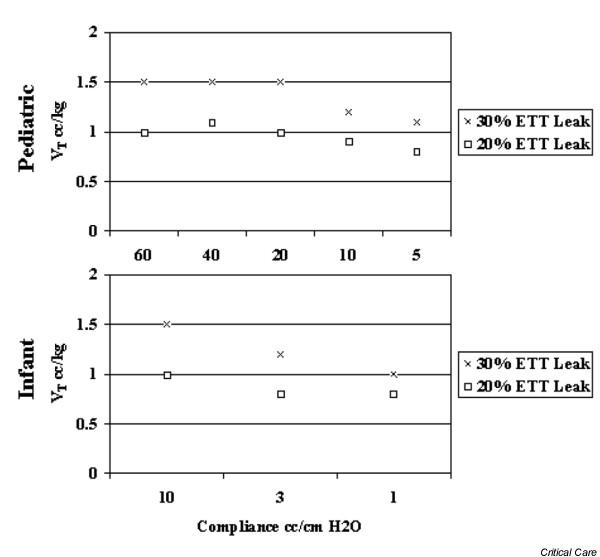
Effect of decreasing lung compliance on the difference between effective tidal volume and tidal volume at the endotracheal tube (ETT) in the infant and pediatric test lung models with 20% and 30% simulated ETT leakage.

In all models, in the absence of ETT leakage the difference between effective *V*_T _and *V*_T _measured at the ETT was less than 10% across the range of lung compliances with a standard deviation of 0–0.2 ml/kg for all sets of measurements. As shown in Fig. 3, however, the agreement between effective *V*_T _and *V*_T _measured at the ETT was poor when a 20% and 30% simulated ETT leak was added in the infant and pediatric test lung models. Under these conditions, the effective *V*_T _was at least 10% higher than that measured at the ETT for all simulated conditions, and the standard deviation was 0.1–0.4 ml/kg for all sets of measurements.

## Discussion

Using well-controlled experimental conditions, we showed that in the absence of ETT leakage, effective *V*_T _approximated the *V*_T _measured at the ETT in the test lung even when lung compliance was poor. As expected, exhaled *V*_T _measured at the ventilator became increasingly inaccurate with poor lung compliance. In the presence of ETT leakage, effective *V*_T _overestimated the *V*_T _measured at the ETT by at least 0.6 ml/kg. It is clear that in the presence of ETT leakage, effective *V*_T _is inaccurate and *V*_T _is most accurately estimated at the airway.

We used an *in vitro *model to manipulate experimental conditions while controlling for all other variables. Accurate measurement of *V*_T _is essential when a low-*V*_T _strategy is used to protect injured lungs as is recommended by the recent ARDS Network study [4]. In the adult lung model, we manipulated the compliance to simulate the lung compliance quartiles reported in the ARDSNet study [4]. Our findings have clinical implications. In agreement with other investigators [1-3], we found that unadjusted *V*_T _measured at the ventilator is highly inaccurate. We found this inaccuracy to increase markedly when lung compliance was abnormal. This means that dual-control automated ventilator modes (for example volume support or pressure-regulated volume control) that make adjustments based on *V*_T _measured at the ventilator might ineffectively ventilate patients with poor lung compliance. Automated ventilator modes should be used with care in critically ill children.

We support the current recommendations of previous investigators [1-3] that *V*_T _should be measured at the ETT in critically ill children receiving mechanical ventilator support. These investigators emphasized the need to measure *V*_T _at the ETT for all children; they did not control for the presence of uncuffed ETTs in their studies or evaluate the effect of leakage. Significant loss of *V*_T _occurs when both ETT leakage and poor lung compliance are present. Although the *V*_T _measured at the ETT may underestimate the actual *V*_T _being delivered in this situation, it is still the best estimation of the tidal volume delivered to the lung. Use of cuffed ETTs to minimize ETT leakage may lead to more accurate measurement of *V*_T _when lung compliance is poor [7]. When ETT leakage is 20% or greater, Main and colleagues [8] reported inconsistent tidal volume delivery and gross overestimation of respiratory compliance and resistance in children.

When ETT leakage is minimal, it seems from our simulated lung models that calculation of effective *V*_T _would give similar readings to *V*_T _measured at the airway, even in small patients. This could potentially negate the need for the addition of sensors at the airway and their associated increase in airway resistance for small ETTs [2]. Unfortunately, ETT leakage is dynamic and dependent on head position. Unless a simple, accurate and continuous means of measuring ETT leakage is available, it is safest to measure *V*_T _at the airway in all mechanically ventilated children. Future studies of *V*_T _measurement accuracy in mechanically ventilated children should control for the degree of ETT leakage.

## Key messages

• 	Previous investigators have emphasized the need to measure tidal volume at the endotracheal tube for all mechanically ventilated children.

• 	When endotracheal leakage is minimal, it would appear from this study using simulated lung models that calculation of effective tidal volume would give similar readings to tidal volume measured at the airway, even in small patients.

• 	Future studies of tidal volume measurement accuracy in mechanically ventilated children should control for the degree of endotracheal tube leakage.

## Competing interests

None declared.

## Abbreviations

ARDS = acute respiratory distress syndrome; ETT = endotracheal tube; FRC = functional residual capacity; PEEP = positive end-expiratory pressure; PIP = peak inspiratory pressure; *V*_T _= tidal volume.

## Figures and Tables

**Table 1:** Experimental conditions for test lung model

Parameter	Infant			Pediatric		Adult	
Simulated weight (kg)	4	7	10	20	31	50	70
ETT internal diameter (mm)	3.0	3.5	4.0	5.0	6.5	7.0	7.5
Tidal volume at 6 ml/kg (ml)	24	42	60	120	186	300	420
PEEP (cmH_2_O)	5	5	5	5	5	5	5
Rate per minute	20	20	20	20	20	12	12
Inspiratory time (s)	1	1	1	1	1	1.2	1.2
FiO_2 _(%)	21	21	21	21	21	21	21
Circuit compliance (ml/cmH_2_O)	1	1	1	1.5	1.5	2.9	2.9
Servo 300 set range	Neonatal			Pediatric		Adult	
CO_2_SMO Plus sensor	Neonatal			Pediatric		Adult	

ETT, endotracheal tube; FiO_2_, fraction of inspired oxygen; PEEP, positive end-expiratory pressure.
